# Cerebrospinal Fluid IL-10 and IL-10/IL-6 as Accurate Diagnostic Biomarkers for Primary Central Nervous System Large B-cell Lymphoma

**DOI:** 10.1038/srep38671

**Published:** 2016-12-07

**Authors:** Yang Song, Wei Zhang, Li Zhang, Wei Wu, Yan Zhang, Xiao Han, Chen Yang, Lu Zhang, Daobin Zhou

**Affiliations:** 1 Department of Hematology, Peking Union Medical College Hospital, Peking Union Medical College & Chinese Academy of Medical Sciences, Beijing, China; 2 Department of Clinical Laboratory, Peking Union Medical College Hospital, Peking Union Medical College & Chinese Academy of Medical Sciences, Beijing, China

## Abstract

Early diagnosis of primary central nervous system lymphoma (PCNSL) represents a challenge, and cerebrospinal fluid (CSF) cytokines may be diagnostic biomarkers for PCNSL. We used an electrochemiluminescence immunoassay to measure interleukin (IL)-10, IL-6, IL-8 and tumor necrosis factor α (TNF-α) in the CSF of 22 B cell PCNSL patients and 80 patients with other CNS diseases. CSF IL-10 was significantly higher in PCNSL patients than in the control group (median 74.7 pg/ml vs < 5.0 pg/ml, *P* < 0.000). Using a CSF IL-10 cutoff value of 8.2 pg/ml, the diagnostic sensitivity and specificity were 95.5% and 96.1%, respectively (AUC, 0.957; 95% CI, 0.901–1.000). For a CSF IL-10/IL-6 cutoff value of 0.72, the sensitivity was 95.5%, and the specificity was 100.0% (AUC, 0.976; 95% CI, 0.929–1.000). An increased CSF IL-10 level at diagnosis and post-treatment was associated with poor Progression free survival (PFS) for patients with PCNSL (*P* = 0.0181 and *P* = 0.0002, respectively). A low diagnostic value for PCNSL was found with CSF IL-8 or TNF-α. In conclusion, increased CSF IL-10 was a reliable diagnostic biomarker for large B cell PCNSL, and an IL-10/IL-6 ratio facilitates differentiation from other conditions, especially a CNS infection.

Primary central nervous system lymphoma (PCNSL) is a rare type of aggressive non-Hodgkin’s lymphoma (NHL) that accounts for ~1% of all lymphomas and 3% of CNS tumors and is predominantly (90–95%) of the diffuse large B-cell lymphoma (DLBCL) subtype^1^. The incidence of PCNSL has increased over the past 20 years, especially in immunocompetent adults^2^,^3^. Early diagnosis of PCNSL represents a challenge. Open surgery or stereotactic biopsy is the standard diagnostic procedure for PCNSL, but reliable early diagnostic biomarkers in blood/CSF samples are needed.

Interleukin (IL)-10 plays a role in lymphoma development by promoting B lymphoma cell proliferation and inhibiting apoptosis^4^,^5^,^6^. Intraocular lymphoma (IOL), a specific type of PCNSL, is characterized by increased IL-10 in vitreous fluid. Elevated IL-10 and an IL-10/IL-6 ratio >1.0 are useful for differentiating between IOL and intraocular infectious diseases; the diagnostic sensitivity is 74–90% and specificity is 75–85%^7^,^8^,^9^,^10^. In 1997, Whitcup’s group first reported increased CSF IL-10 in two cases of PCNSL^11^, and a few studies with a small sample size also reported a correlation between CSF IL-10 and PCNSL, suggesting that CSF IL-10 may act as a biomarker of PCNSL^12^,^13^,^14^,^15^. At the same time, the sensitivity and specificity of CSF IL-10 for diagnosing PCNSL varies across reports, and the false positivity of CSF IL-10 has not been clearly interpreted, especially given that IL-10 is also elevated in various conditions other than PCNSL, such as CNS infection and autoimmune diseases.

In this study, we recruited control patients with a diagnosis other than PCNSL to explore the diagnostic ability of CSF IL-10 and IL-10/IL-6 to identify PCNSL. A standardized laboratory method with good performance, i.e., the electrochemiluminescence immunoassay (ECLIA), was used to determine the cytokine levels. In addition, studies suggest that increased serum IL-8 and tumor necrosis factor α (TNF-α) levels are correlated with poor prognosis in systemic DLBCL^16^,^17^,^18^. Therefore, we also assessed the diagnostic value of CSF IL-8 and TNF-α in PCNSL patients. This study aimed to identify reliable biomarkers for initial screening that can facilitate early diagnosis of PCNSL.

## Results

### Patient Characteristics

We identified 22 consecutive B cell PCNSL patients (21 cases of pathologically confirmed diffuse large B-cell lymphoma and one case with CSF confirmed to have a mature B-cell origin by flow cytometry), and all patients were Chinese (Tables 1 and 2). The following were the 5 control groups (N = 80): (1) 14 patients with CNS inflammatory disease (Neuro-infla), including multiple sclerosis, CNS vasculitis, neuropsychiatric systemic lupus erythematosus, and anti-NMDA receptor encephalitis; (2) 13 patients with CNS infection (Neuro-infec), including viral encephalitis, encephalopyosis, meningitis and toxoplasmosis; (3) 5 patients with CNS demyelinating disease (DD); (4) 7 patients with other brain tumors (OBTs), including B-cell acute lymphoblastic leukemia (ALL), small B-cell lymphoma, T-cell lymphoma and other hematopoietic tumors involving the CNS; and (5) 41 cases of high-risk, aggressive systemic NHL involving the CNS (S-NHL) (Table 1 and Supplementary Tables 1 and 2).

All PCNSL patients received methotrexate-based induction chemotherapy followed by intensive chemotherapy or radiotherapy according to the age of the patient and the response to treatment. The treatment response was evaluated according to the IPCG criteria^19^. The median follow-up of patients was 11.0 months (3.0–18.0 months); only patients with complete data of the prognosis were included in the survival analysis (data from 5 patients were censored). There were 5 events (PD or death).

### CSF Cytokine Profile in controls and PCNSL patients at the time of diagnosis

**Figure 1:**
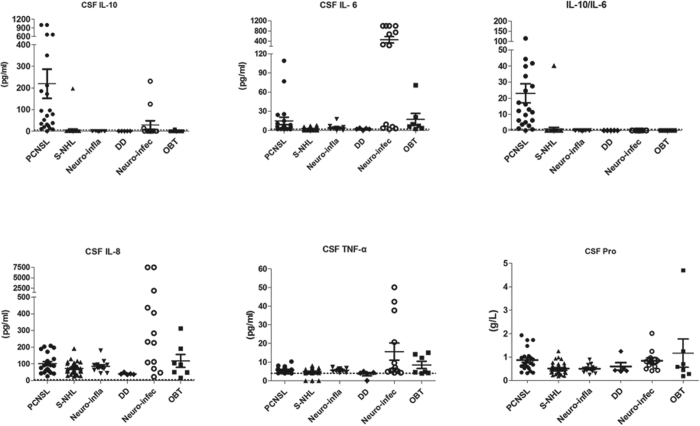
Scatter diagram for the CSF protein and cytokine profile of PCNSLs and other disease types. Dashed lines indicate the detection lower limits for CSF IL-10, IL-6, IL-8, and TNF-α and 0.72 for the IL-10/IL-6 ratio.

As Table 1 (also Fig. 1) clearly shows, the CSF IL-10 level was uniformly increased in all PCNSL patients, and there was an IL-10/IL-6 ratio greater than 0.72, except in 1 case (case 7). While in the control group, the CSF protein and pre-inflammatory cytokine (IL-6, IL-8, and TNF-α) levels might increase in some patients, but the CSF IL-10 level was below the lower limit of detection (<5 pg/ml), except in 5 cases. Three cases had CNS infection, and one had T-cell lymphoma involving the leptomeninges. Although the patients’ CSF IL-10 levels were increased, the IL-10/IL-6 ratios were less than 0.16. The remaining 1 case was an S-NHL patient, who temporally diagnosed with primary testicular lymphoma (PTL) without evidence of CNS involvement after extensive investigations, including routine CSF testing, fundal examination and head-enhanced computed tomography. His pre-chemotherapy CSF IL-10 level and IL-10/IL-6 ratio were 197 pg/ml and 40.2, respectively, and his CSF IL-10 level dropped below the detection limits after 1 cycle of aggressive chemotherapy (CHOP + methotrexate 1 g/m^2^).

### Clinical Characteristics and CSF examination in PCNSL patients

Table 2 showed all characteristics of patients with PCNSL. Notably, the CSF IL-10 level was very high (>1,000 pg/ml) in two patients (cases 8 and 9). One had meningeal involvement, and the other had spinal cord PCNSL involving the thoracic and lumbar segments; both were positive for lymphoma cells in the CSF, which was confirmed by flow cytometry and cytology (Table 2).

To evaluate the interdependence of CSF cytokines and clinical features in PCNSL patients, pairwise Spearman’s rank coefficients were calculated. Overall, the CSF IL-10 level was correlated with increased CSF protein (r 0.587, *P* = 0.004), the numbers of lesions (r 0.521, *P* = 0.013)and Prognostic Scoring System for PCNSL(r 0.625, *P* = 0.002), but the level was not related to the CSF pressure or serum lactate dehydrogenase (sLDH) in PCNSL patients (Table 3).

### ROC Curve Analysis

**Figure 2:**
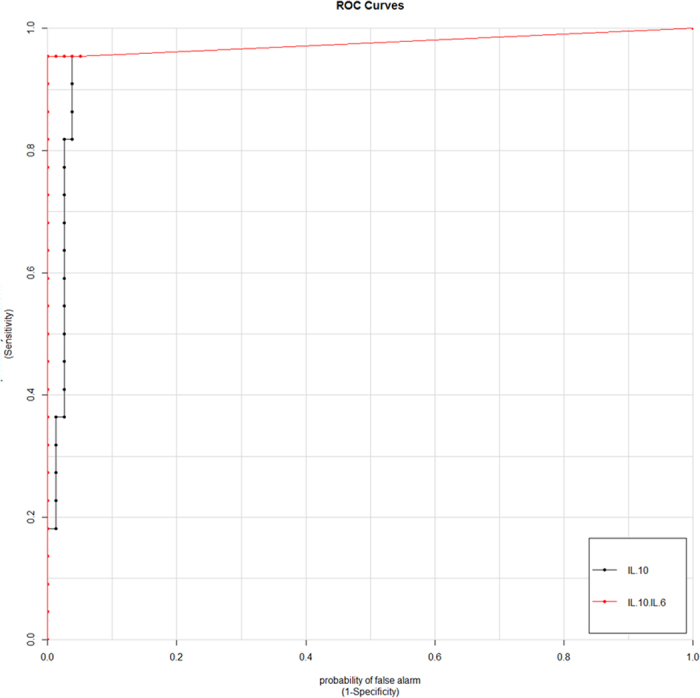
Receiver-operator characteristic (ROC) curves of the CSF IL-10 and IL-10/IL-6. IL-10: sensitivity 95.5%, specificity 96.1% at 8.2 pg/ml (AUC, 0.957; 95% CI, 0.901–1.000); IL-10/IL-6: sensitivity 95.5%, specificity 100.0% at 0.72 (AUC, 0.976; 95% CI, 0.929–1.000). AUC, *area under the curve* and CI, *confidence interval.*

The ability of CSF cytokines to identify PCNSL was assessed by ROC curves. At a CSF IL-10 cutoff level of 8.2 pg/ml, the diagnostic sensitivity and specificity for PCNSL were 95.5% and 96.1%, respectively (AUC, 0.957; 95% CI, 0.901–1.000). For an IL-10/IL-6 ratio cutoff level of 0.72, the sensitivity and specificity were 95.6% and 100.0% (AUC, 0.976; 95% CI, 0.929–1.000), respectively (Fig. 2). Cross-validated area under the ROC curves(cvAUC) of the CSF IL-10 and IL-10/IL-6 were 0.955 and 0.975, respectively.

(Supplementary Fig. S1 and Supplementary Fig. S2). Low to moderate diagnostic value was obtained with CSF IL-6, IL-8, TNF-α, or protein (Supplementary Fig. S3). The only case of PTL with an elevated CSF IL-10 level, as mentioned above, was not included due to suspected secondary CNS lymphoma.

### Correlation between the CSF IL-10 level and treatment response

**Figure 3:**
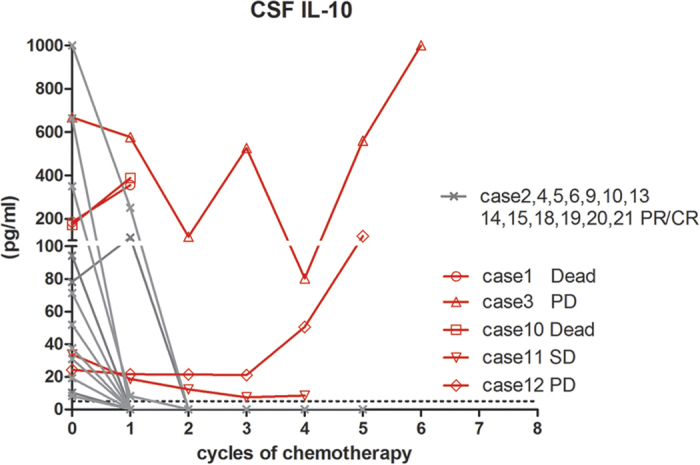
Dynamic changes in the CSF IL-10 in PCNSL patients (n = 17). The post-chemotherapy CSF level of IL-10 decreased below the detection lower limit in 12 patients and was accompanied with disease remission (CR/PR). In contrast, five cases (cases 1, 3, 10, 11, and 12) had persistent IL-10 elevation; all reached SD/PD (2 of cases died). *Dashed lines indicate the lower limits of detection.*

The relationship between the CSF IL-10 level and diseases status in PCNSL patients was explored by dynamic monitoring in 17 cases (Fig. 2). The post-chemotherapy CSF IL-10 level dropped to below the lower limit of detection in 12 patients. The decline in the CSF IL-10 level was accompanied by disease remission (CR/PR). The other 5 cases had persistent IL-10 elevation despite chemotherapy. Two of them (cases 1 and 10) experienced early death (<6 months) and the remaining cases (cases 3, 11, and 12) reached SD/PD at the final follow-up (after 5–7 cycles of chemotherapy), which was confirmed by magnetic resonance imaging (MRI) (Fig. 3).

**Figure 4:**
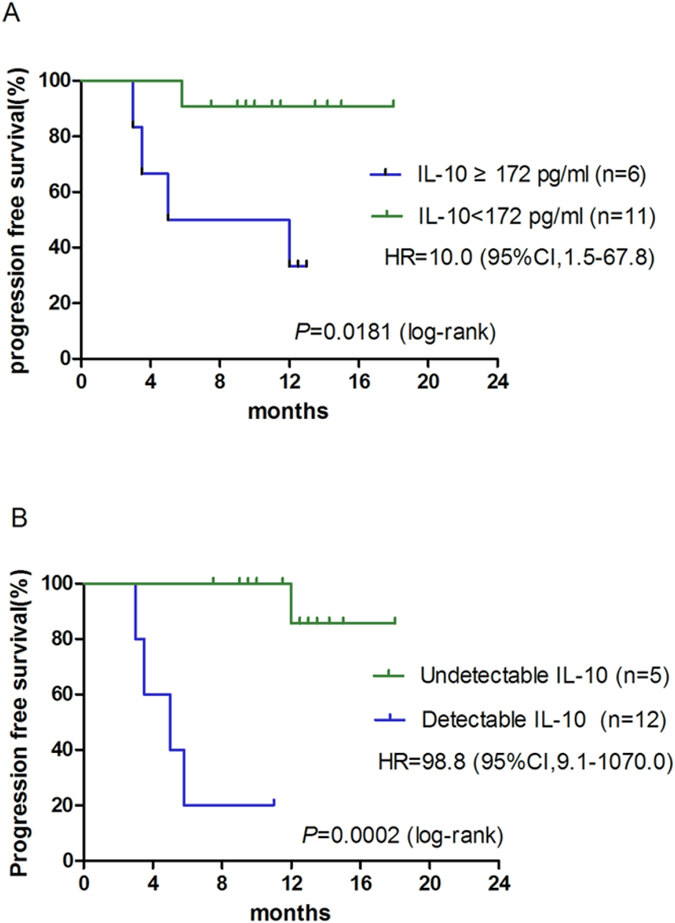
PFS of PCNSL patients according to the cerebrospinal fluid interleukin (IL-10) concentration at diagnosis (**A**) and post-treatment (**B**). Post-treatment is defined as after 2 cycles of chemotherapy. PFS, *progression free survival*; HR, *hazard ratio* and CI, *confidence interval.*

We also examined the impact of the CSF IL-10 concentrations on the outcomes of PCNSL patients (n = 17). Higher CSF IL-10 levels at both the beginning and after the 2nd cycle of chemotherapy were significantly associated with poor PFS (*P* = 0.0181 and *P* = 0.0002, respectively) (Fig. 4).

## Discussion

The electrochemiluminescence immunoassay (ECLIA) procedure is a method based on the solid-phase sandwich immunoassay^20^. It was performed using commercially available laboratory analytical instruments with the advantages of superior accuracy and repeatability of cytokine measurements^21^,^22^,^23^. The procedure was rapid and automated, which was especially convenient for monitoring dynamic changes and minimizing the artificial bias. In this study, we demonstrated that elevated CSF IL-10 and CSF IL-10/IL-6 have high sensitivity and specificity for diagnosing large B cell PCNSL via ECLIA.

Until recently, only four relatively large sample size studies have evaluated the diagnostic value of CSF IL-10 in PCNSL. The reported sensitivity of CSF IL-10 in diagnosing PCNSL is 65.4–94.7% with a specificity of 88.9–100%^12^,^13^,^14^,^15^. The subtle differences from our results may be due to methodological differences. However, the role of IL-6 and the IL-10/IL-6 ratio were not determined in the other studies. Based on the results of the present study^13^,^15^, their roles were under-estimated, at least in distinguishing between PCNSL and intracranial infections. First, in Rubenstein’s study, IL-10 was not sufficiently specific to differentiate between patients with PCNSL versus CNS infections/inflammation (CSF IL-10 was positive in 4 out of 12 cases with meningitis). Serial Combination of IL-10 and CXCL13 was used to improve the diagnostic specificity to 99.3%, at the cost of a poor sensitivity (50%)^13^. Second, small B-cell lymphoproliferation with leptomeningeal involvement can elevate CSF IL-10 levels in 7 out of 17 cases, which was not the case for IL-10/IL-6, except in 2 patients with DLBCL transformation and concurrent PCNSL in one study^24^. This is inconsistent with our observation that IL-10 can be elevated in conditions other than PCNSL, such as meningitis and T-cell lymphoma with leptomeningeal involvement. Moreover, our study further indicated the role of the IL-6. A cut-off value for IL-10/IL-6 of 0.72, which yielded diagnostic specificity of 100% with preserved sensitivity (95.5%). We speculate that CNS infection/inflammation can also increase CSF IL-10, a well-known inhibitory cytokine, which presumably results from immune cell secretion as a feedback response to pro-inflammatory cytokines, including IL-6, in the presence of infection/inflammation^25^. Therefore, the CSF IL-10/IL-6 ratio has an important role in the differential diagnosis of PCNSL.

The CSF IL-10 level may also reflect the severity of PCNSL. In our study, an elevated CSF level of IL-10 was associated with a shorter progression free survival in PCNSL patients, which is consistent with previous reports^12^. In addition, the CSF IL-10 level correlated with elevated CSF protein and multiple brain lesions, and it was highly consistent with the therapeutic response. Therefore, the CSF IL-10 level may reflect the tumor burden or an active disease state. Notably, the CSF IL-10 level was especially high (>1,000 pg/ml) in two patients with meningeal/spinal cord and CSF involvement, which is similar to early reports^11^,^15^,^26^. Therefore, we hypothesized that the CSF IL-10 level may be associated with the lesion location and that the CSF IL-10 level may increase more often in patients with positive CSF cytology and meningeal/spinal cord involvement. Furthermore, longitudinal IL-10 measurement may be much more reliable if the lesion location is a deterministic factor of the CSF IL-10 level.

The CSF IL-10 levels were also associated with the lymphoma histology based on the available literature, including the new findings of our current study. Thus far, significant CSF IL-10 correlation with CNSL (primary or secondary) was only observed in patients with the DLBCL subtype. To the best of our knowledge, only one study with other histological types of PCNSL (two cases of T-cell PCNSL) has been published, and the authors did not report an elevated CSF IL-10 level^12^. In our study, the CSF IL-10 level was also negative in 2 B-cell ALL cases and 1 small B-cell lymphoma case with CNS involvement. The CSF IL-10 level mildly increased, but the IL-10/IL-6 ratio was 0.16 (<0.72), in one case of T-cell lymphoma involving the leptomeninges. In addition, a study reported that IL-10 was not an accurate biomarker for detecting leptomeningeal involvement in small B-cell lymphoproliferation^24^. All the above evidence suggested that IL-10 may be a specific biomarker for the DLBCL subtype of CNSL.

It is unclear whether CSF IL-10 is secreted by tumor cells or by cells within the tumor niche. One study reported that the CSF levels of IL-10 in PCNSL patients were correlated with infiltration of tumor associated macrophages (TAM) and TAM cells expressing IL-10, suggesting that CSF IL-10 may be primarily secreted by TAMs^27^. However, PCNSL tumor cells were also found to express IL-10^12^,^13^. Furthermore, T-cell PCNSL and other brain tumors/metastases also had TAM infiltration, but the CSF IL-10 level was negligible in these cases. In addition, the CSF level of IL-10 was significantly correlated with the therapeutic response in PCNSL patients. Therefore, we predicted that CSF IL-10 is more likely to be secreted by tumor cells in PCNSL than from the tumor microenvironment.

The molecular mechanism behind elevated CSF IL-10 in PCNSL remains unclear. Recent studies indicated that a high frequency (>75%) of MYD88 L265P mutations (an adaptor protein mediating toll-like receptor and interleukin-1 signaling) in the DLBCL subtype of PCNSL may be an initiator of disease. Additionally, most MYD88 L265P-positive patients have CD79B or CARD11 mutations (B cell receptor signaling pathway)^28^,^29^,^30^. In addition, the constitutive activation of the nuclear factor (NF)- κB pathway is a hallmark of B cell PCNSL^31^. MYD88 L265P, CD79B and CARD11 mutations can cause excessive activation of the NF-κB pathway, eventually resulting in increased secretion of IL-10^32^. Meanwhile, as an inhibiting inflammatory cytokine, IL-10 contributed to the inhibitory tumor microenvironment, which promoted tumorigenesis and progression^4^,^25^. Further study is required to confirm this hypothesis.

In summary, the combination of elevated CSF IL-10 and IL-10/IL-6 levels is a reliable diagnostic biomarker for PCNSL and superior to traditional parameters used in initial screening for PCNSL patients. And that the CSF IL-10 level may reflect the severity and therapeutic response of the diseases. Meanwhile, the CSF IL-10/IL-6 ratio is an important differential diagnosis parameter, especially for CNS infections. The study sample size limited the power of our conclusions and these results should be validated in a distinct patient cohort. Additionally, accumulation of studies is warranted to give definitive conclusion on the impact of CSF IL-10 in prognosis of CNS lymphoma.

## Methods

### Populations

The study participants were recruited from January 2015 to March 2016 at Peking Union Medical College Hospital. The study groups were consecutively enrolled, newly diagnosed or relapsed PCNSL patients. All patients had a histological diagnosis, and none had a history of immunodeficiency. Serial CSF samples were collected before the start of initial chemotherapy, before each cycle of chemotherapy and at relapse/progression in a subset of PCNSL patients.

Meanwhile, patients with diagnoses other than CNS lymphoma were included for comparison, such as patients with CNS inflammation, infection, demyelination, acute leukemia or NHL involving CNS, and high-risk systemic NHL involving the CNS (Burkitt’s lymphoma, lymphoblastic lymphoma, or Double-hit DLBCL; NHL with the involvement of any of the following locations: epidural, spine proximal, eye sockets, sinuses, kidneys, adrenal glands, bone, bone marrow, testes, breast, female genitalia; or NHL with ≥2 extranodal lesions and elevated serum lactate dehydrogenase levels). Exclusion criteria included age <12 years and traumatic CSF collection.

This study was approved by the ethical committee of Peking Union Medical College Hospital and registered at Chinese Clinical Trial Registry (ChiCTR-DDD-15007568)^33^. Informed consent was obtained from all patients. All methods and procedures in this study met the Declaration of Helsinki.

### Sample Preparation and Electrochemiluminescence Immunoassay

CSF samples were collected from all patients by lumbar puncture without frozen preservation. Routine biochemical examination, including white cell counts, cytological examinations, and flow cytometry (FCM), was performed. CSF samples (1 ml) were centrifuged (10 min at 500 × g at 18 °C). Supernatant was collected, and the IL-10, IL-6, IL-8, and TNF-α levels were measured with an electrochemiluminescence immunoassay (ECLIA) analyzer according to the manufacturer’s instructions (Siemens Immulite 1000 and its corresponding IL-10, IL-6, IL-8, and TNF-α detection kits). The procedure was fully automated and took 60 minutes. Detection ranges were as follows: CSF IL-10 5–1,000 pg/ml; IL-6 2–1,000 pg/ml; IL-8 5–7,500 pg/ml; and TNF-α 4–1,000 pg/ml. No sample dilution was performed for values exceeding the maximal detection limit. Values below the lower detection limits were treated as 0. The intra- and inter-assay coefficients of variation for each cytokine were <5.6% and 1.1–8.9% for IL-10, <11.0% and 4.0–11.0% for IL-6, <4.9% and 2.8–9.5% for IL-8, and <6.5% and 2.7–7.8% for TNF-α.

### Statistical Analysis

Differences between the groups were analyzed using the Kruskal-Wallis H test (Graphpad Prim 5.0). Spearman’s correlation was performed to evaluate the interdependency of CSF cytokines and clinical features (SPSS 18.0). The ability of each CSF cytokine level to identify PCNSL was assessed with receiver-operator characteristic (ROC) curves, which included the sensitivity, specificity, cut-off value, area under the curve (AUC), 95% confidence interval (CI), and then was evaluated by *K*-fold cross-validation using R 3.2.2 pROC package. The predictive value of the CSF cytokine concentrations in the progression free survival (PFS) of patients with PCNSL was assessed with a log rank test (Graphpad Prim 5.0). The PFS was calculated from the date of CSF sample collection to the date of death and date of progression/recurrence or the date of the last follow-up. All tests were 2 tailed, and p < 0.05 was considered statistically significant.

## Additional Information

**How to cite this article**: Song, Y. *et al*. Cerebrospinal Fluid IL-10 and IL-10/IL-6 as Accurate Diagnostic Biomarkers for Primary Central Nervous System Large B-cell Lymphoma. *Sci. Rep.* **6**, 38671; doi: 10.1038/srep38671 (2016).

**Publisher's note:** Springer Nature remains neutral with regard to jurisdictional claims in published maps and institutional affiliations.

## Supplementary Material

Supplementary Figures and Tables

## Figures and Tables

**Table 1:** CSF protein and cytokine profile (mean and range) in PCNSL cases and other disease types.

Diagnosis	N	Gender	Age *(years)*	Pro *(g/L)*	IL-10 *(pg/ml)*	IL-6 *(pg/ml)*	IL-8 *(pg/ml)*	TNF-a *(pg/ml)*	IL-10/IL-6
PCNSL	22	8 M/14 F	57 (27–70)	0.75 (0.33–1.93)*^1^	*74.7 (*<5.0–1000)*^2^	*5.2 (*<2.0–109.0)*^3^	89.5 (25.0–208.0)	5.7 (4.5–10.3)	12.6 (0.0–114.9)*^4^
S-NHL	41	24 M/17 F	49 (20–78)	0.47 (0.18–1.25)	<5.0 (<5.0–197.0)	2.4 (<2.0–8.2)	61.5 (28.0–129.0)	5.0 (<4.0–7.1)	0.0 (0.0–40.2)
Neuro-infla	14	3 M/11 F	34 (20–70)	0.50 (0.24–0.89)	<5.0	3.2 (<2.0–17.8)	78.0 (43.0–116.0)	5.7 (4.8–6.7)	0.0 (0.0–0.0)
DD	5	2 M/3 F	50 (27–59)	0.45 (0.38–1.24)	<5.0	2.2 (<2.0–3.9)	39.0 (35.0–50.0)	4.1 (<4.0–5.0)	0.0 (0.0-.0.0)
Neuro-infec	13	8 M/5 F	33 (22–71)	*0.80 (0.49–2.01)*^*#1*^	<5.0 (<5.0–231.0)	*271.0 (*<2.0*–1000)*^#2^	*232.0 (22.0–7500)*^#3^	*5.8 (4.3–50.1)*^#4^	0.0 (0.0–0.2)
OBT	7	4 M/3 F	53 (39–64)	0.58 (0.19–4.70)	<5.0 (<5.0–7.9)	*7.3 (6.4–70.8)*^△1^	96.0 (17.0–312.0)	4.8 (4.2–15.1)	0.0 (0.0–0.1)

*1* P* < 0.01 vs S-NHL; *2* P* < 0.0001 vs all other disease types; *3* P* < 0. 01 vs S-NHL, *4 *P* < 0.0001 vs all other disease types, except OBT(*P* < 0.001). #1 *P* < 0.01 vs S-NHL; #2 *P* < 0.0001 vs S-NHL, *P* < 0.01 vs DD; #3 *P* < 0.001 vs S-NHL, *P* < 0.0001 vs DD; and #4 *P* < 0.01 vs DD. ^△^1 *P* < 0.01 vs S-NHL. (Kruskal-Wallis H test after adjusting for multiple comparison).

Abbreviations: PCNSL, primary cent*ral nervous system lymphoma*; S-NHL, *systematic NHL*; Neuro-infla, *neuro-inflammatory diseases*; DD, *demyelinating diseases*; Neuro-infec, *neuro-infection*; OBT, *other brain tumors*; and Pro, CSF protein.

**Table 2:** Clinical characteristics and CSF examination in all PCNSL patients.

Case	Age/sex	Diagnosis	Lesion location	PS	sLDH *(U/L)*	Status	CSF							Treat- ment	Resp onse
							Pro *(g/L)*	IL-10 *(pg/ml)*	IL-6 *(pg/ml)*	IL-8 *(pg/ml)*	TNFa *(pg/ml)*	IL-10/IL-6	Cytology/FCM		
1	61/F	PCNSL	basal ganglia, thalamus, ventricle	4	191	Rec	0.86	*185.0*	10.7	91.0	5.3	*17.3*	−/−	EA/RT	Dead
2	64/M	PCNSL	basal ganglia, temporal lobe	2	134	Rec	0.97	*94.2*	8.3	97	6.3	*11.3*	−/−	MTX	CR
3	61/F	PCNSL	lateral ventricle, corpus mamillare	2	215	New	0.67	*668.0*	24.3	41.0	5.9	*24.5*	−/−	R+MTX	SD
4	43/F	PCNSL	lateral ventricle, frontal lobe, corpus callosum	4	255	New	0.33	*10.3*	3.5	47.0	5.5	*2.9*	−/−	MTX	CR
5	27/F	PCNSL	basal ganglia	3	182	New	0.75	*19.1*	0.1*	63.0	6.0	*191.0*	−/−	R+MTX	CR
6	58/M	PCNSL	basal ganglia	4	183	New	1.72	*78.1*	109.0	172.0	5.7	*0.7*	−/−	MTX	PR
7	55/F	PCNSL	basal ganglia, corpus callosum	3	191	New	0.31	<*5.0*	2.6	69.0	4.1	*0.0*	−/−	MTX	NA
8	61/M	PCNSL	basal ganglia, temporal lobe, meningeal	4	373	New	1.73	*>1000*	76.9	208.0	10.3	*13.0*	+/+	R+MTX	NA
9	54/F	PCNSL	spinal cord	4	469	New	0.89	*>1000*	8.7	203.0	7.7	*114.9*	+/+	R+MTX	PR
10	69/F	PCNSL	basal ganglia, corpus callosum, lateral ventricle	4	159	New	1.04	*172.0*	5.1	197.0	6.7	*33.7*	−/−	R+MTX	Dead
11	62/M	PCNSL	temporal lobe	2	167	New	0.68	*33.7*	6.7	92.0	6.8	*5.0*	−/−	R+MTX	PR
12	57/F	PCNSL	temporal lobe	3	174	New	0.74	*24.3*	1.3*	75.0	4.5	*18.7*	−/−	R+MTX	SD
13	45/M	PCNSL	temporal lobe	3	175	New	0.59	*37.5*	6.0	117.0	4.4	*6.3*	−/−	MTX	CR
14	65/F	PCNSL	frontal lobe, parietal lobe	3	245	New	1.47	*664.0*	15.0	129.0	8.1	*44.3*	−/−	R+MTX	PR
15	70/F	PCNSL	frontal lobe, temporal lobe	4	334	New	1.02	*30.9*	22.5	189.0	4.0	*1.2*	−/−	MTX	CR
16	70/F	PCNSL	right temporal lobe	3	149	New	0.61	*95.8*	2.3	48.0	4.0	*41.7*	−/−	R-MTX	NA
17	55/M	PCNSL+IOL	right eye, frontal lobe, temporal lobe	3	259	New	1.93	*211.0*	5.3	60.0	5.9	*39.8*	−/−	R-MTX	NA
18	63/F	PCNSL+IOL	left eye, frontal lobe, corpus callosum	4	324	New	0.52	*51.9*	3.2	103.0	5.0	*1.8*	−/−	MTX	CR
19	48/F	PCNSL+IOL	left eye, basal ganglia, temporal lobe	2	270	New	0.98	*350*	4.3	88.0	6.0	74.6	−/−	MTX	CRu
20	37/M	IOL	binoculus	1	216	New	0.52	*71.3*	2.9	56.0	5.6	*24.6*	−/−	MTX	CR
21	37/F	IOL	right eye	1	170	New	0.39	*8.2*	2.2	43.0	5.1	*3.7*	−/NA	MTX	CR
22	38/M	IOL	left eye	1	216	New	0.34	*12.3*	2.1	25.0	4.9	*6.2*	−/−	MTX	NA

Abbreviations: PS, *Eastern Cooperative Oncology Group performance status*; sLDH, *serum lactate dehydrogenase*; Pro, *Protein;* R, *Rituximab*; MTX, *High dose methotrexate (5 g/m*^*2*^); E, *Etoposide*; A, *Cytarabine*; RT, *radiotherapy*; PD, *progressive disease*; SD, *stable disease*; PR, *partial remission*; CR, *complete remission*; CRu, *CR/unconfirmed*; FCM, *flow cytometry;* −*, negative;* +*, positive; and* NA, *not available*.

^*^Below detection limits and calculated according to the sample luminous intensity.

**Table 3:** Correlation analyses for the CSF IL-10 and Clinical characteristics in PCNSL patients.

	CSF IL-10	
	r	*p*
Age	0.409	0.059
ECOG PS	0.146	0.550
sLDH	0.282	0.204
***CSF Pro***	***0.587***	***0.004***
***Lesion numbers***	***0.521***	***0.013***
CSF pressure	0.384	0.078
***PSS for PCNSL***	***0.625***	***0.002***

Moderate-to-high correlation (r >0.5), Significant correlation (*P* < 0.05). r, *Spearman’s rank correlation coefficient; p, P value*.

Prognostic Scoring System for PCNSL(0–5):*older than 60 years-of-age; ECOG 2–4; elevated serum LDH; increased CSF protein; and involvement of deep brain regions.*
