# Supplementary material for: Cerebrospinal Fluid IL-10 and IL-10/IL-6 as Accurate Diagnostic Biomarkers for Primary Central Nervous System Large B-cell Lymphoma
Source: Sci Rep. 2016 Dec 7;6:38671. doi: 10.1038/srep38671 (PMC5141427; doi:10.1038/srep38671)
Supplement: Supplementary Figures and Tables [file srep38671-s1.doc]

**Cerebrospinal Fluid IL-10 and IL-10/IL-6 as** **Accurate Diagnostic Biomarkers for Primary Central Nervous System Large B-cell Lymphoma**

Yang Song^1^, Wei Zhang^1^, Li Zhang^2^, Wei Wu ^2^, Yan Zhang ^1^, Xiao Han ^1^, Chen Yang^1^, Lu Zhang^1^, Daobin Zhou^1^


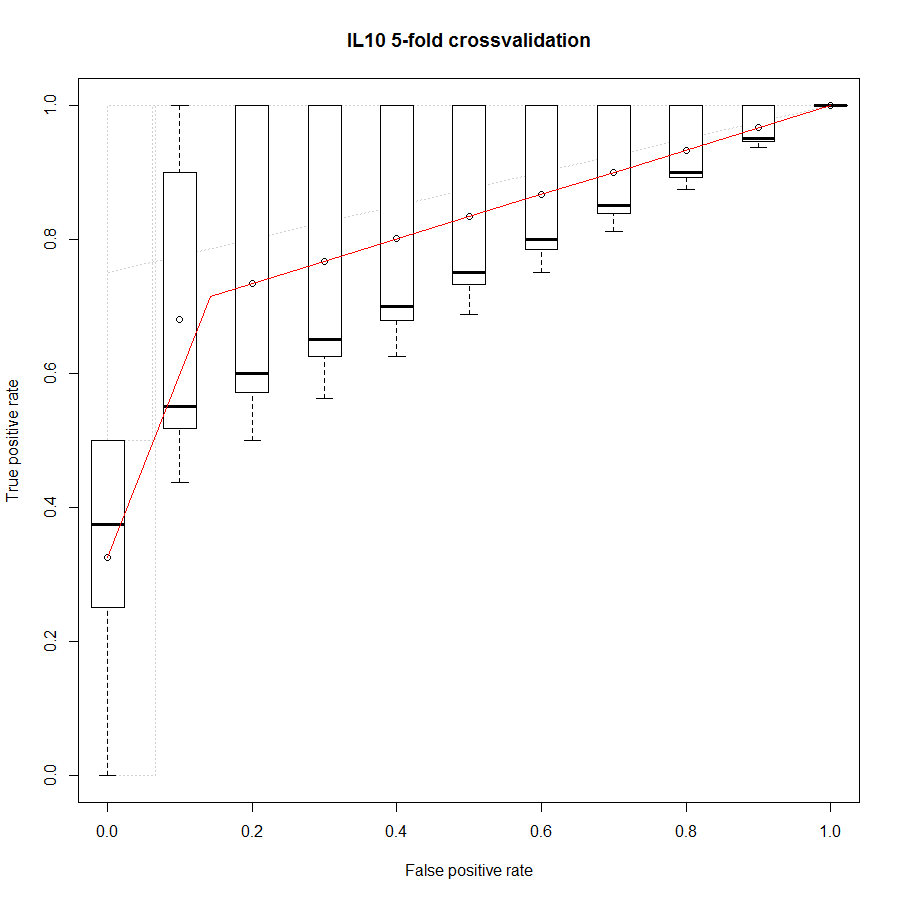


Supplementary Fig S1. Cross-validated area under the Receiver-operator characteristic (ROC) curves of the CSF IL-10. CSF IL-10 cvAUC=0.955.

cvAUC, *cross-validated area under the ROC curve.*


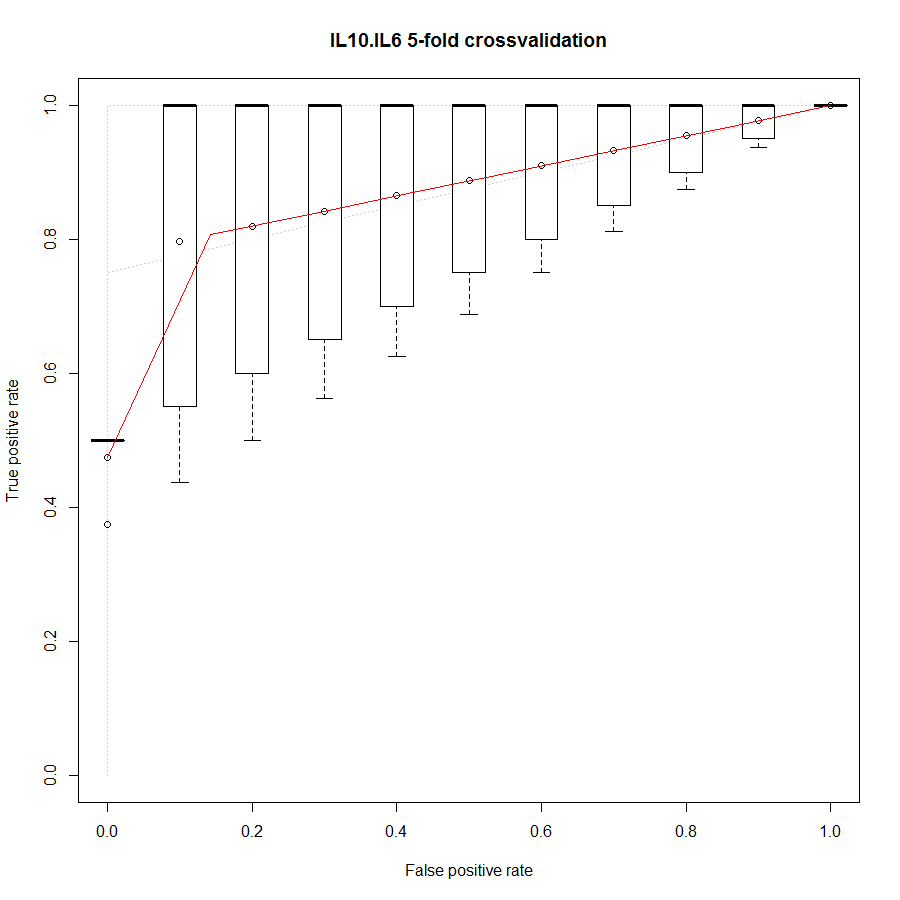


Supplementary Fig S2. Cross-validationed area under the Receiver-operator characteristic (ROC) curves of the CSF IL-10/IL-6. CSF IL-10/IL-6 cvAUC=0.975.

cvAUC, *cross-validated area under the ROC curve.*

*
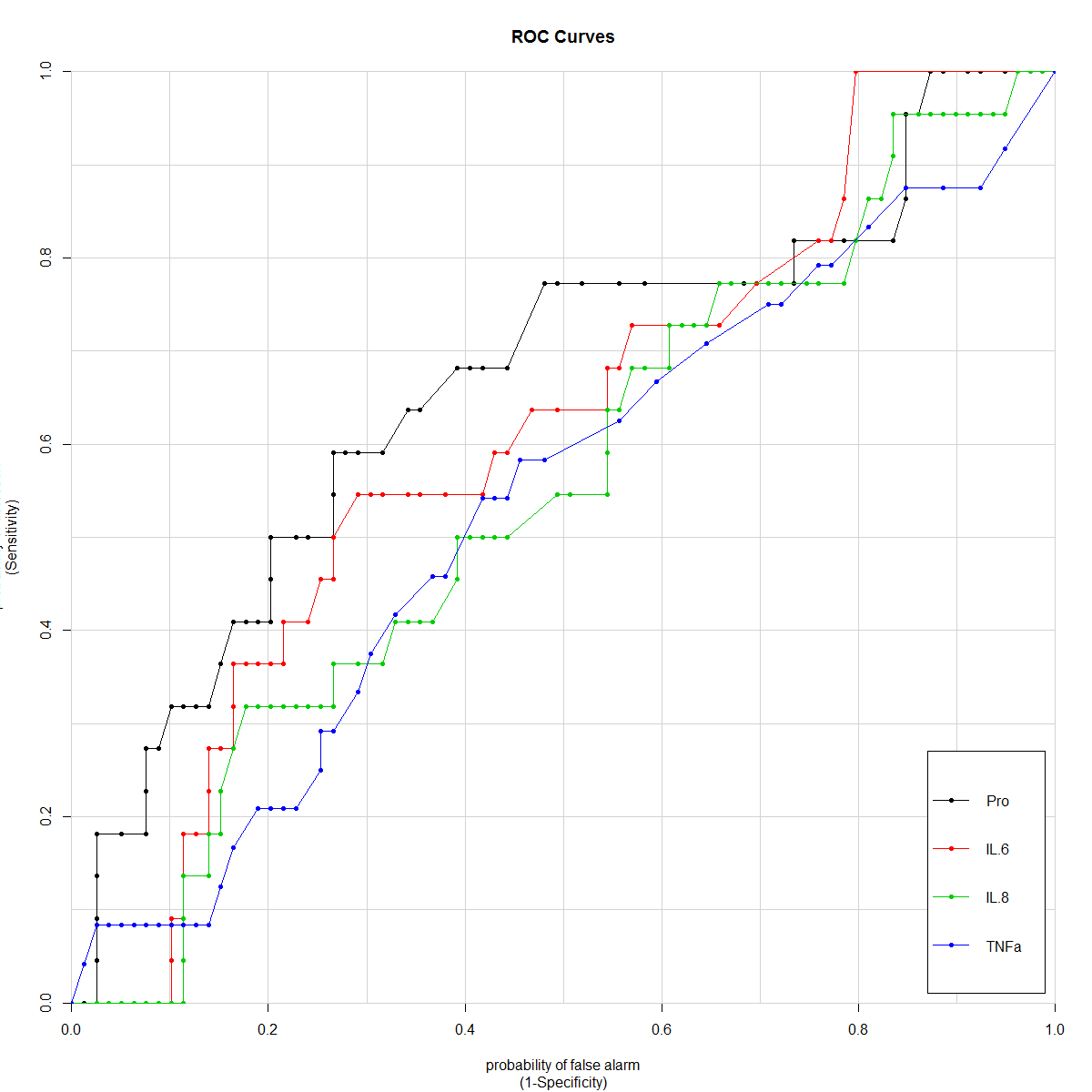
*

Supplementary Fig S3. Receiver-operator characteristic (ROC) curves of the CSF cytokines. Pro: sensitivity 59.1%, specificity 74.0% at 0.67 g/L (AUC, 0.668; 95% CI, 0.527-0.808); IL-6: sensitivity 54.6%, specificity 70.1% at 5.1 pg/ml (AUC, 0.609; 95% CI, 0.479-0.739); IL-8: sensitivity 31.8%, specificity 83.1% at 117 pg/ml (AUC, 0.556; 95% CI, 0.422-0.691); and TNF-a: sensitivity 59.1%, specificity 57.1% at 5.3 pg/ml (AUC, 0.660; 95% CI, 0.407-0.681). AUC, *area under the curve* and CI, *confidence interval.*

Supplementary Table S1. Clinical characteristics and CSF profile in patients with other CNS dieases.

| **case** | **Sex** | **Age** | **diagnosis** | **Pro**  *(g/L)* | **IL-10**  *(pg/ml)* | **IL-6**  *(pg/ml)* | **IL-8**  *(pg/ml)* | **TNFa**  *(pg/ml)* |
| --- | --- | --- | --- | --- | --- | --- | --- | --- |
|  | | | **neuro-inflammation** |  | | | | |
| 1 | M | 34 | multiple sclerosis | 0.42 | <5.0 | 3.0 | 44.0 | 5.7 |
| 2 | M | 78 | multiple sclerosis | 0.45 | <5.0 | 17.8 | 116.0 | 6.5 |
| 3 | F | 34 | multiple sclerosis | 0.56 | <5.0 | 4.1 | 71 | 4.30 |
| 4 | F | 70 | NPSLE | 0.50 | <5.0 | 3.0 | 81.0 | 5.8 |
| 5 | F | 32 | NPSLE | 0.30 | <5.0 | <2.0 | 43.0 | 6.5 |
| 6 | F | 27 | NPSLE | 0.35 | <5.0 | 2.2 | 94.0 | 7.3 |
| 7 | F | 20 | NPSLE | 0.52 | <5.0 | 6.8 | 95.0 | 6.7 |
| 8 | F | 31 | NPSLE | 0.24 | <5.0 | <2.0 | 63.0 | not done |
| 9 | F | 34 | NPSLE | 0.89 | <5.0 | 3.2 | 75.0 | 5.1 |
| 10 | F | 31 | CNS vasculitis | 0.49 | <5.0 | 32 | 92.0 | 5.9 |
| 11 | F | 65 | CNS vasculitis | 0.66 | <5.0 | 2.6 | 83.0 | 5.1 |
| 12 | F | 42 | CNS vasculitis | 0.38 | <5.0 | 3.8 | 75.0 | 4.9 |
| 13 | F | 48 | Plasma cell granuloma | 0.51 | <5.0 | 5.1 | 75.0 | 4.2 |
| 14 | M | 27 | anti-NMDA receptor encephalitis | 0.71 | <5.0 | 4.0 | 180.0 | 5.5 |
|  | | | **neuro-infection** |  | | | | |
| 1 | M | 71 | Viral  encephalitis | 0.91 | <5.0 | 9.5 | 226.0 | 5.5 |
| 2 | M | 22 | Viral  encephalitis | 0.81 | <5.0 | 5.7 | 112.0 | 5.1 |
| 3 | M | 61 | encephalopyosis | 0.84 | <5.0 | 3.1 | 22.0 | 9.5 |
| 4 | F | 33 | encephalopyosis | 0.86 | <5.0 | >1000.0 | 283.0 | 20.7 |
| ***5*** | M | 69 | encephalopyosis | 0.49 | <5.0 | 236.0 | 109.0 | 4.3 |
| 6 | M | 65 | encephalopyosis | 0.731 | <5.0 | >1000.0 | 232.0 | 5.8 |
| 7 | F | 26 | meningoencephalitis | 0.65 | <5.0 | >1000.0 | >7500.0 | 5.7 |
| ***8*** | ***F*** | ***27*** | [***tubercular***](javascript:void(0);) [***meningitis***](javascript:void(0);) | ***2.01*** | ***231.0*** | ***>1000.0*** | ***>7500.0*** | ***42.3*** |
| ***9*** | ***F*** | ***25*** | ***meningitis*** | ***0.97*** | ***13.9*** | ***673.0*** | ***1913.0*** | ***50.1*** |
| ***10*** | ***M*** | ***57*** | ***meningitis*** | ***1.24*** | ***125.0*** | ***751.0*** | ***405.0*** | ***37.7*** |
| 11 | F | 33 | meningitis | 0.59 | <5.0 | 271 | 436 | 7.1 |
| 12 | M | 31 | Toxoplasmosis | 0.41 | <5.0 | 2.0 | 47.0 | 4.8 |
| 13 | M | 41 | Toxoplasmosis | 0.43 | <5.0 | 3.5 | 71.0 | 4.3 |
|  | | | **Demyelinating Diseases** |  | | | | |
| 1 | M | 52 | Demyelinating Diseases | 1.24 | <5.0 | 3.9 | 50.0 | 5.0 |
| 2 | F | 50 | Demyelinating Diseases | 0.52 | <5.0 | 2.2 | 37.0 | 4.4 |
| 3 | F | 48 | Demyelinating Diseases | 0.43 | <5.0 | <2.0 | 39.0 | <4.0 |
| 4 | M | 27 | Demyelinating Diseases | 0.45 | <5.0 | 2.1 | 35.0 | 4.1 |
| 5 | F | 59 | Demyelinating Diseases | 0.38 | <5.0 | 3.1 | 40.0 | 4.0 |
|  | | | **Other brain tumurs** |  | | | | |
| ***1*** | ***M*** | ***46*** | ***ENKTCL involving leptomeninges*** | ***4.70*** | ***7.9*** | ***70.8*** | ***312.0*** | ***15.1*** |
| 2 | F | 60 | Small B-cell lymhpma involving CNS | 0.58 | <5.0 | 6.4 | 105.0 | 14.2 |
| 3 | M | 53 | ECD involving CNS | 0.19 | <5.0 | 10.1 | 17.0 | 12.3 |
| 4 | M | 64 | B-ALL involving CNS | 0.7 | <5.0 | 7.3 | 49.0 | 4.8 |
| 5 | F | 39 | B-ALL involving CNS | 0.45 | <5.0 | 21.1 | 51.0 | 4.2 |
| 6 | 1 | 54 | DLBCL involving endocranium | 0.28 | <5.0 | 2.4 | 96.0 | 4.0 |
| 7 | 0 | 43 | DLBCL involving endocranium | 0.25 | <5.0 | 3.8 | 192.0 | 4.6 |

Abbreviations: NPSLE,*neuropsychiatric systemic lupus erythematosus*; ENKTCL,*extranodal*

*NK/T-cell lymphoma*; B-ALL, *B cell acute lymphoblastic leukemia.*

Supplementary Table S2. Clinical characteristics and CSF profile in S-NHL patients.

| **case** | **Sex** | **Age** | **diagnosis** | **Risk factors** | **Pro**  *(g/L)* | **IL-10**  *(pg/ml)* | **IL-6**  *(pg/ml)* | **IL-8**  *(pg/ml)* | **TNFa**  *(pg/ml)* |
| --- | --- | --- | --- | --- | --- | --- | --- | --- | --- |
| 1 | M | 76 | DLBCL(ABC) | eye socket | 0.59 | <5.0 | <2.0 | 56.0 | <4.0 |
| 2 | F | 77 | DLBCL(ABC) | womb | 0.63 | <5.0 | 2.5 | 110.0 | 4.2 |
| 3 | F | 59 | DLBCL(GCB) | breast | 0.47 | <5.0 | 4.7 | 98.0 | 5.1 |
| 4 | F | 41 | DLBCL(GCB) | uterine adnexa | 0.57 | <5.0 | <2.0 | 85.0 | 5.3 |
| 5 | F | 61 | DLBCL(ABC) | oophoron | 0.28 | <5.0 | <2.0 | 40.0 | 4.9 |
| 6 | M | 49 | DLBCL(ABC) | spine proximal | 0.61 | <5.0 | 2.5 | 98.0 | 5.7 |
| 7 | M | 50 | DLBCL(GCB) | spine proximal | 0.52 | <5.0 | <2.0 | 101.0 | 5.0 |
| 8 | M | 49 | DLBCL(ABC) | spine proximal | 0.35 | <5.0 | 2.2 | 84.0 | 6.0 |
| 9 | M | 45 | DLBCL(ABC) | bone | 0.50 | <5.0 | 5.1 | 65.0 | 4.5 |
| 10 | F | 31 | DLBCL(GCB) | bone | 0.18 | <5.0 | 2.4 | 97.0 | 7.1 |
| 11 | M | 39 | DLBCL(ABC) | marrow | 0.31 | <5.0 | 3.0 | 25.0 | 6.8 |
| 12 | F | 63 | DLBCL(ABC) | marrow | 0.22 | <5.0 | 3.0 | 32.0 | 5.6 |
| 13 | M | 72 | DLBCL(ABC) | kidney | 0.47 | <5.0 | 3.9 | 53.0 | 5.1 |
| 14 | M | 33 | DLBCL(ABC) | kidney | 0.36 | <5.0 | <2.0 | 28.0 | 4.4 |
| 15 | F | 78 | DLBCL(ABC) | adrenal gland | 0.26 | <5.0 | 2.7 | 59.0 | 4.6 |
| 16 | M | 45 | DLBCL(ABC) | adrenal gland | 0.59 | <5.0 | 6.0 | 117.0 | 4.4 |
| 17 | M | 68 | DLBCL(ABC) | adrenal gland | 0.34 | <5.0 | 2.2 | 55.0 | 4.8 |
| 18 | F | 45 | DLBCL(ABC) | EL ≥ 2 | 0.44 | <5.0 | <2.0 | ND | 4.8 |
| 19 | M | 32 | DLBCL(GCB) | EL ≥ 2 | 0.20 | <5.0 | <2.0 | 101.0 | ND |
| 20 | F | 38 | DLBCL(GCB) | EL ≥ 2 | 0.56 | <5.0 | 2.2 | 51.0 | 8.0 |
| 21 | M | 43 | DLBCL(ABC) | EL ≥ 2 | 0.70 | <5.0 | 4.1 | 57.0 | 6.1 |
| 22 | M | 67 | DLBCL(GCB) | epilepsy | 0.37 | <5.0 | <2.0 | 72.0 | 7.0 |
| 23 | F | 56 | DLBCL(T-rich) | adrenal gland | 0.93 | <5.0 | 3.4 | 48.0 | <4.0 |
| 24 | F | 52 | DLBCL(T-rich) | bone | 0.35 | <5.0 | 2.2 | 71.0 | 5.0 |
| 25 | F | 34 | DLBCL(double hit) | marrow | 0.45 | <5.0 | 2.4 | 75.0 | 4.9 |
| 26 | F | 48 | DLBCL(double hit) | breast | 0.76 | <5.0 | 6.4 | 29.0 | 4.7 |
| 27 | F | 43 | DLBCL（triple-hit） | triple-hit | 1.25 | <5.0 | 3.8 | 192.0 | 4.6 |
| 28 | M | 58 | FL-IIIb | kidney | 0.63 | <5.0 | 2.3 | 49.0 | 4.9 |
| 29 | F | 20 | Burkitt lymphoma | Burkitt | 0.98 | <5.0 | 8.2 | 92.0 | <4.0 |
| 30 | M | 52 | Burkitt lymphoma | Kidney, marrow | 0.61 | <5.0 | 2.5 | 106.0 | 5.2 |
| 31 | F | 30 | Burkitt lymphoma | Oophoron, | 0.24 | <5.0 | <2.0 | 52.0 | 4.8 |
| 32 | M | 64 | PTL(DLBCL) | testis | 0.45 | <5.0 | <2.0 | 53.0 | 5.4 |
| 33 | M | 49 | PTL(DLBCL) | testis | 0.49 | <5.0 | <2.0 | 111.0 | 5.2 |
| ***34*** | ***M*** | ***56*** | ***PTL(DLBCL)*** | ***testis*** | ***0.46*** | ***197.0*** | ***4.9*** | ***63.0*** | ***5.5*** |
| 35 | M | 44 | ENKTCL-nosel | paranasal sinus | 0.92 | <5.0 | 5.0 | 62.0 | 4.1 |
| 36 | M | 48 | ENKTCL-nosel | paranasal sinus | 0.45 | <5.0 | 2.3 | 36.0 | 5.1 |
| 37 | M | 46 | ENKTCL-nosel | paranasal sinus | 0.45 | <5.0 | 7.3 | 57.9 | 5.3 |
| 38 | M | 56 | PTCL-NOS | EL ≥ 2 | 0.32 | <5.0 | 2.3 | 129.0 | 5.5 |
| 39 | M | 58 | ALCL(ALK-) | EL ≥ 2 | 0.73 | <5.0 | 2.5 | 53.0 | 4.1 |
| 40 | F | 32 | ALCL(ALK-) | epilepsy | 0.60 | <5.0 | <2.0 | 61.0 | 6.0 |
| 41 | M | 43 | TLBL | TLBL | 0.37 | <5.0 | <2.0 | 61.0 | ND |

### Abbreviations: DLBCL(ABC/GCB), *diffuse large B-cell lymphoma( activated B-cell like subtype /germinal center B-cell like subtype)*; FL, *follicular lymphoma*; PTL, *primary testicular lymphoma*; ENKTCL,*extranodal NK/T-cell lymphoma*; PTCL-NOS, *pheripheral T-cell lymphoma not otherwise specipied*; ALCL, *anaplastic large cell lymphoma*; TLBL, *T-cell lymphoblastic lymphoma*; ECD, *Erdheim-Chester Disease*; EL ≥ 2*,* *extranodal lesions* ≥ 2 and *elevated LDH;* ND*,not done.*
